# Management of critically ill COVID-19 patients: challenges and affordable solutions

**DOI:** 10.11604/pamj.2021.38.270.28689

**Published:** 2021-03-16

**Authors:** Carlo Emanuele Caresia

**Affiliations:** 1 Department of Anesthesia and Intensive Care, Careggi Teaching Hospital, Florence, Italy

**Keywords:** COVID-19, severe pneumonia, non-invasive ventilation, solutions

## To the editors of the Pan African Medical Journal

I read with great interest the paper of Metogo and colleagues about the critically ill COVID-19 patients in Africa [1]. The authors stressed some persistent challenges of the anaesthesiologist-intensivist physicians due to lack or insufficient Intensive Care Units (ICU) beds and mechanical ventilators and paucity of ICU physicians and nurses. As Italian intensivist, with some experiences in Africa (the last ones in South Sudan and Angola, with the Italian NGO Doctors with Africa CUAMM) and with experience with critically ill COVID-19 patients, I would like to suggest some affordable solutions that could be adopted in many African hospitals.

The first challenge raised by the authors is the shortage of Intensive Care Unit (ICU) beds and of mechanical ventilators. One solution could be to create temporary respiratory units with skilled personnel and basically equipped to manage patients with severe pneumonia [2]. During the first wave of COVID-19, in Italy it was a widespread use of mechanical ventilation, which rapidly overwhelmed the capacity of ICUs. To face this problem, in my hospital it was developed a respiratory unit inside the Department of Infectious Diseases, where a group of intensivists took care of the patients with severe pneumonia due to COVID-19. We were equipped with pulse oximeters, an ultrasound machine, oxygen, and a complete set of devices to provide respiratory support: Hi Flow Nasal Cannula, Helmets, portable ventilators for non-invasive ventilation, in order to avoid, whenever possible, the endotracheal intubation. In my opinion, this experience can be transferred and adapted in many African hospitals, providing a basic and not expensive equipment: at least pulse oximeters, bottles of oxygen, and a few respiratory devices. Among these, I suggest the helmet, an economic and easy-to-use device: it delivers high flow oxygen with a Continuous Positive Air Pressure (CPAP) that produces a Positive End Expiratory Pressure (PEEP), which allows the lungs to remain inflated and, ultimately, improves the oxygen exchange [3]. Due to shortage of oxygen in many African countries, I advise a model with a special valve (Venturi valve), which requires only 15 litres per minute of fresh oxygen but provides a total flow of 60 litres per minute with fraction of oxygen of 4% and a PEEP up to 10 cm H_2_O. Another useful device is the Boussignac System, consisting of a mask and a special valve, which works with the same mechanism of CPAP and PEEP [4]. I also recommend, wherever possible, an ultrasound machine as versatile diagnostic tool at the bedside: for lungs (to evaluate the extension of the interstitial pneumonia) [5], for heart (to investigate heart failure, for example in suspicion of pulmonary embolism), and for peripheral vascular system (to detect thrombosis).

The second challenge addressed by the authors is about the paucity of African anaesthesiologist-intensivist physicians and anaesthetist-ICU nurses. In my experiences in Africa, I learned that skilled people are more important than sophisticated instruments: therefore, I could suggest creating feasible courses to allow health workers to acquire more skills. In the contest of COVID-19, I consider two especially important skills. The first is a diagnostic skill: to evaluate at first glance the patient and to recognize if he is going to worse before he becomes critical. Clinical evaluation and a pulsi oximeter at the bedside allow not only to recognize the current state of the patients but even predict the evolution, through simples scores that match clinical findings and the saturation of oxygen. One of the easiest is the ROX index (Ratio of Oxygen saturation): it is the ratio of the saturation (measured by pulse oximetry) /FIO2 to respiratory rate [6]. Another score, more complete, is the NEWS (National Early Warning Score), which include oxygen saturation plus the main clinical signs (respiratory rate, systolic blood pressure, cardiac pulse, temperature and consciousness) [7]. The second is a manual skill: to perform simple physical manoeuvre in order to improve the respiratory function of the patients. Among these, turning the patients in prone position is of paramount importance: prone position enhances oxygenation through several mechanism that concur to improve the distribution of alveolar ventilation and blood flow [8,9]. Based on my experience and according to the literature, I recommend developing a local protocol to applicate the prone position early, daily and safely.

The current pandemic has been teaching us that it is crucial to share our knowledge and our findings. Combining my African and Italian experiences, I am convinced that we can successfully face these big challenges concentrating our efforts on training health workers and on looking for suitable and affordable technical solutions.
